# The behavioral pattern of patients with frontotemporal dementia during the COVID-19 pandemic

**DOI:** 10.1017/S104161022000109X

**Published:** 2020-06-10

**Authors:** Maki Suzuki, Maki Hotta, Aki Nagase, Yuki Yamamoto, Natsuho Hirakawa, Yuto Satake, Yuma Nagata, Takashi Suehiro, Hideki Kanemoto, Kenji Yoshiyama, Etsuro Mori, Mamoru Hashimoto, Manabu Ikeda

**Affiliations:** 1 Department of Behavioral Neurology and Neuropsychiatry, United Graduate School of Child Development, Osaka University, Osaka, Japan; 2 Department of Psychiatry, Course of Integrated Medicine, Graduate School of Medicine, Osaka University, Osaka, Japan

## How the coronavirus 2019 pandemic affects patients with frontotemporal dementia?

Due to cognitive impairment, people with dementia in general are more likely to have a greater risk for coronavirus disease 2019 (COVID-19) exposure. At present, both caregivers and care receivers are undergoing considerable stress than usual (Japanese Psychogeriatric Society, http://www.rounen.org). Japanese government as well as other countries’ one has strongly encouraged to stay at home except for essential activities, to wear face masks in public, to wash hands frequently, to maintain social distance between people, and so on. For people with dementia, it may be difficult to understand and adapt to the dramatic change of unusual social situation and their memory problems make instructions problematic to remember (Alzheimer’s disease International, https://www.alz.co.uk/news/adi-releases-position-paper-on-covid-19-and-dementia).

Frontotemporal dementia (FTD) is known to have distinct clinical features from Alzheimer’s disease (AD), developing behavioral and language symptoms with relatively preserved memory function (Ducharme *et al.*, 2020; Neary *et al.*, 1998; Rascovsky *et al.*, 2011). Patients with FTD particularly tend to show distinctive unusual behaviors such as disinhibition, loss of social awareness, overeating, perseverative and stereotyped behavior, and impulsivity which are often antisocial (Ikeda, 2007; Neary *et al.*, 1998; Rascovsky *et al.*, 2011). Indeed, patients with FTD showed a significant higher rate of law violation as compared to AD patients (Shinagawa *et al.*, 2017). Therefore, we hypothesize that patients with FTD would have difficulty adapting to the drastic changes in lifestyle caused by the COVID-19 outbreak, and their carers might be under unique stress.

Here, we report the results of our study of investigating the difficulties of patients with FTD in relatively mild stages and their carers under the COVID-19 pandemic, and provide information that may help to support individualized care for patients with FTD.

## Patients with FTD and AD and their carers undertook semi-structured interviews of preventive actions against COVID-19

We aimed to include home-dwelling patients with FTD and AD and their carers. Patients with FTD who are regularly followed in the memory clinic of Department of Psychiatry, Osaka University Hospital, Osaka, Japan, were selected and met the following criteria: (1) those with relatively preserved basic activities of daily living (ADL) (more than 4 in the Physical Self-Maintenance Scale (PSMS) score; Lawton and Brody, 1969); one FTD patient was scored 4 due to her cerebral palsy and included in this survey); (2) those with a reliable informant; and (3) those who were able to provide informed content. Patients with AD were selected to match those with FTD group for age, sex, and PSMS score. The diagnosis of each dementia was established according to the international consensus criteria (Gorno-Tempini *et al.*, 2011; McKhann *et al.*, 2011; Neary *et al.*, 1998; Rascovsky *et al.*, 2011). All patients undertook routine neuropsychological and neuropsychiatric examinations, including the Mini-Mental State Examination (MMSE) (Folstein *et al.*, 1975), Clinical Dementia Rating (CDR) (Hughes *et al.*, 1982), and PSMS. The PSMS is a 6-item scale that evaluates patients’ ability to perform basic ADL (toileting, feeding, grooming, dressing, physical ambulation, and bathing). Scores range from 0 to 6, with higher scores indicating better functioning. Thus, 12 patients with FTD (4 males and 7 females; mean age = 71.8 ± 5.9 years; PSMS = 5.6 ± 0.6; CDR = 0.9 ± 0.4; MMSE = 15.6 ± 7.4) and 12 patients with AD (4 males and 7 females; mean age = 72.4 ± 6.8 years; PSMS = 5.7 ± 0.6; CDR = 0.7 ± 0.2; MMSE = 22.9 ± 3.2) and their carers participated in this study. The FTD group included two patients with behavioral variant FTD (bvFTD), one with progressive non-fluent aphasia, and nine with semantic dementia (SD).

The survey was started after Japan’s April 7 Emergency Declaration and continued approximately 2 weeks to outpatients and/or their carers of our dementia clinic in face to face or televisits. Semi-structured interviews of preventive actions against COVID-19 were conducted by one of the medical staffs including neuropsychiatrists, neuropsychologists, occupational therapists, and geriatric nurse practitioners. Questions in this study included (1) Does the patient stay at home except for essential reasons? (“Staying home”), (2) Does the patient wear a face mask when he/her go out? (“Wearing a mask”), (3) Does the patient wash his/her hands when he/her come home? (“Washing hands”), and (4) Does the patient maintain physical distance between himself/herself and others? (“Social distance”). The carers were asked to answer “Yes, by himself/herself (0),” “Do after being told to do (1),” “No, even if I told to do (2),” and “Don’t know” to each question. The number scores (0, 1, 2) were given for response categories. In addition, the examiner interviewed the caregiver about the daily life of the patient. A Mann–Whitney *U*-test was performed to compare the scores of the two groups (*p* < 0.05 was considered significant). “Don’t know” responses (two in “Social distance”, respectively, for each group) were not considered in the analyses. This study was performed in line with the principles of the Declaration of Helsinki. Approval was granted by the Ethics Committee of Osaka University.

## Results showed patients with FTD were more difficult to take preventive measures against COVID-19 than AD

FTD group scored higher than AD group on “Washing hands” (*p* < 0.05) and “Social distance” (*p* < 0.01). In addition, there was a trend toward significance between two groups for “Staying home” (*p* < 0.1), with higher score for patients with FTD. Remaining one (“Wearing a mask”) did not differ between the groups (*p* = 0.89). Many FTD patients did not wash their hands spontaneously and required instructions by carer. This tendency was more prominent in “Social distance.” A considerable proportion of FTD patients showed difficulty in maintaining physical distance with other people even after they were requested to do so by carers. For “Staying home”, several FTD patients did not follow the carers’ words and went out for habitual activities (see below for example).

## Representative examples of FTD patients’ action of preventive measures

Case 1 was a 69-year-old right-handed male with SD of left-hemisphere predominant involvement (2 years after onset of symptoms; PSMS = 5). The patient went to pachinko pinball parlor, which is one of his habitual activities, for several times. According to his wife, “It was impossible to stop him when he decided to go out.” It seems that the patient does not understand the current situation at all. For example, when he went for a walk to the park nearby, he called to his wife and said, “There were no people inside the park. Why?” (“Staying home”). The patient is reluctant to wear a face mask but when his wife put it inside his bag, he usually wears it (“Wearing a mask”). When the wife stood apart from the person in front at the register counters in a supermarket, the patient forced her to move forward. Even if the wife pointed out mark lines on floor and explained about social distancing, he continued to step closer (“Social distance”).

Case 2 was a 70-year-old right-handed female with SD of right-hemisphere predominant involvement (2 years after onset of symptoms; PSMS = 6). The patient goes shopping or walking as usual (Japanese government currently does not regulate going out for both activities) (“Staying home”). She wears a face mask spontaneously but never wash her hands with a soap. Her daughter said, “When I told her to do hand washing, she asked me why. It seems that she cannot understand that her hands are unclean without visible dirt” (“Washing hands”). The patient does not understand what the coronavirus is and even does not be aware of some kind of disease is under outbreak situation.

## Why patients with FTD showed difficulty in following social distances, washing hands, and staying home?

In the present study, patients with FTD showed significantly more difficulties in keeping social distances than those with AD. It would be very difficult for patients with FTD to keep more than 2 m from others in a row in front of the register counters in supermarkets and to avoid crowded spaces such as gyms and public transports because FTD patients frequently develop socially inappropriate behaviors (disinhibition) and a lack of understanding or indifference to the feelings of others (loss of empathy). Moreover, most FTD patients showed the difficulties to understand the meaning of “social distance.”

In addition, patients with FTD showed significant difficulties in washing hands and staying home to compare with AD patients. FTD patients become increasingly inflexible and often adopt a fixed daily routine (stereotyped behavior). To introduce new lifestyle habit such as washing hands and staying home instead of each patient’s unique daily routine might be difficult by family carers.

Another intriguing finding was that only mask wearing behavior did not differ between patients with FTD and AD. To understand the importance of prevention measures, it is required to understand the general reason behind them beyond what they actually look like. Patients with FTD are known to have difficulty in understanding abstract meaning from concrete experiences (Hashimoto *et al.*, 2011). Indeed, some FTD patients had trouble to understand that hand washing is the act of removing invisible germs and lines taped across supermarket floor are for physical distancing. This idea should also apply to mask wearing. However, since all carers wear mask when they go out, tendency to get easily influenced by visual stimuli might facilitate the FTD patients to do mask wearing (stimulus-bound behavior/environmental dependency syndrome) (Ikeda, 2007).

Similar to FTD patients, some AD patients showed difficulties in wearing a face mask, washing hands, and keeping social distances probably due to their memory impairment. At least, they could do these just after they were told to do so by carers.

## Conclusion

The present results revealed that patients with FTD were more difficult to take preventive measures against COVID-19 (social distance, washing hands, and staying home) than those with AD. To keep social distance was of more difficult, many FTD patients did not follow carers’ instructions. Some preventive measures (such as wearing face masks) may possible to facilitate by utilizing their behavioral symptom of stimulus-bound behavior/environmental dependency syndrome. The behavioral problems of patients with FTD under COVID-19 would lead to increased caregivers’ burden due to worries about patients’ behaviors and fear of resulting infection, and fatigue of repeating the same explanations to the patients. During the current COVID-19 pandemic, special attention to the caregiving issues in FTD is an urgent need as well as, or more than those in AD, at least in relatively mild stage.
